# Picosecond infrared laser driven sample delivery for simultaneous liquid-phase and gas-phase electron diffraction studies

**DOI:** 10.1063/4.0000159

**Published:** 2022-09-16

**Authors:** Zhipeng Huang, Meghanad Kayanattil, Stuart A. Hayes, R. J. Dwayne Miller

**Affiliations:** 1 Max Planck Institute for the Structure and Dynamics of Matter, Luruper Chaussee 149, 22761 Hamburg, Germany; 2 Departments of Chemistry and Physics, University of Toronto, 80 St. George Street, Toronto, Ontario M5S 1H6, Canada

## Abstract

Here, we report on a new approach based on laser driven molecular beams that provides simultaneously nanoscale liquid droplets and gas-phase sample delivery for femtosecond electron diffraction studies. The method relies on Picosecond InfraRed Laser (PIRL) excitation of vibrational modes to strongly drive phase transitions under energy confinement by a mechanism referred to as Desorption by Impulsive Vibrational Excitation (DIVE). This approach is demonstrated using glycerol as the medium with selective excitation of the OH stretch region for energy deposition. The resulting plume was imaged with both an ultrafast electron gun and a pulsed bright-field optical microscope to characterize the sample source simultaneously under the same conditions with time synchronization equivalent to sub-micrometer spatial resolution in imaging the plume dynamics. The ablation front gives the expected isolated gas phase, whereas the trailing edge of the plume is found to consist of nanoscale liquid droplets to thin films depending on the excitation conditions. Thus, it is possible by adjusting the timing to go continuously from probing gas phase to solution phase dynamics in a single experiment with 100% hit rates and very low sample consumption (<100 nl per diffraction image). This approach will be particularly interesting for biomolecules that are susceptible to denaturation in turbulent flow, whereas PIRL–DIVE has been shown to inject molecules as large as proteins into the gas phase fully intact. This method opens the door as a general approach to atomically resolving solution phase chemistry as well as conformational dynamics of large molecular systems and allow separation of the solvent coordinate on the dynamics of interest.

## I. INTRODUCTION

Most chemistry, and all biology, occurs in the solution phase. A long-standing challenge has been to ascertain the effect of the solvent on the reaction coordinate and better understand the distinctive features of solution phase homogeneous chemistry in relation to gas phase or heterogeneous chemistry involving surfaces. We need to understand how different solvents affect the reaction pathways. The present work focuses on a novel method to generate simultaneously isolated gas phase molecules, up to biological systems, as well as nanoscale thick liquid domains to directly separate solvent effects in a single sample delivery system to specifically address this issue. In this regard, the solvent effects on reaction coordinates and structural dynamics can now be observed at the atomic level of detail by taking advantage of the recent advent of ultrabright electron and x-ray sources. These sources have made it possible to directly observe atomic motions during the defining moments of structural transitions.^1–6^ In particular, these sources have made it possible to observe the far from equilibrium motions and highly anharmonic coupling between vibrational modes that leads to the formation of the key reaction modes directing chemistry.^1,3,4,7–9^ It is this enormous reduction in dimensionality that occurs during barrier crossing events that lead to the transferability of chemical reaction mechanisms, or the named reactions, exploited in synthetic chemistry.^4,7–11^ The methods for atomically resolving these motions principally involve time resolved diffraction of either electron or x-ray structural probes. Most of these studies have been directed at solid state chemistry^1,3,4,7–11^ and unimolecular reactions in the gas phase. There has also been some seminal work in the study of solution phase processes,^12–20^ but these studies are more limited in scope. There is currently a gap in the relative number of studies of solution phase chemistry or homogeneous chemistry, which is unfortunate given the significance of the role of solvent in directing chemical processes. Time resolved x-ray diffraction methods have been developed to address this gap, where standard methods using flow cells or jets can be used for sample exchange.^12–15^ However, to separate the effect of solvent, independent experiments are needed on the same gas phase system under the same conditions, which is not generally feasible as two completely different sample delivery systems are involved. There are also issues related to subtraction of thermal effects on background scattering from the host solvent, which limits the resolution over solid-state studies of reaction dynamics. This limitation largely reflects the differences in relative contribution of background scattering to the diffraction signal of interest. For single crystals, all molecules participate, whereas only the solute, typically a small molar fraction, is undergoing structural changes in solution phase studies. It is necessary to use as high concentration as possible in solution phase studies to minimize this background scattering. The greatest limitation is peak power. In pushing the time resolution to the femtosecond domain, the requirements for high excitation to get above scattering contributions from background invariably lead to competing multiphoton absorption at the peak powers typically used (>100 GW/cm^2^), which bring into question the initial state preparation in defining the reaction pathway.^21,22^ These two technical issues of removing background scattering and peak power limitations need extensive method development, as well as new means of analysis, to approach the linear, nonpertubative, regime probed by all optical methods. Progress in this regard is hindered by the limited access to X-ray Free Electron Lasers (XFELs), which are the only x-ray sources capable of 100 fs time resolution with sufficient brightness to follow the primary processes in solution phase reaction dynamics.

The alternative is to exploit ultrabright electron sources, which opened the study of atomically resolved reaction dynamics.^1–4,7–9^ These are readily available tabletop sources with comparable time resolution and spatial resolution to XFELs. The much larger scattering cross section of electrons relative to x-rays enables the use of extremely thin samples, on the 10–100 nm scale, and correspondingly, lower peak powers to attain the same fraction of excitation yet remain in the linear regime. This condition is essential to avoid peak power and multiphoton artifacts. The main technical limitation in the use of electron sources for solution phase studies is attaining the prerequisite 100 nm thin pathlengths with flow to allow sample exchange between excitation pulses using pump–probe protocols.^16,17,19,20,23–25^ This problem has been addressed using high pressure virtual nozzles to make ultrathin jets, which have led the way toward atomically resolved solution phase studies.^23,24^ The hydrodynamic parameters to generate stable 100 nm thick liquid jets in the high vacuum needed for electron studies depend strongly on viscosity and surface tension. So far, this sample delivery method has only been demonstrated for aqueous systems, arguably the most important case. It will be interesting to see how general this method can be made and whether large molecules such as proteins can survive the turbulent flow conditions without denaturation. None of these methods allow a direct comparison of isolated molecules and solution phase under identical conditions as needed to directly determine the role of solvent on molecular dynamics.

The present work addresses the need for the development of a general method of introducing both solution phase and gas phase systems suitable for use with electron probes to atomically resolve molecular dynamics. Here, it is important to keep in mind that there has been enormous progress in following reaction dynamics through a number of different all optical methods, from conventional fs transient absorption to coherent multidimensional spectroscopic methods. These methods cover the complete spectral range from THz to vacuum ultraviolet (VUV) and soft x-rays. The different spectroscopies have different degrees of sensitivity to specific types of motion or involvement of electronic states in reaction dynamics. The associated structural dynamics still need to be inferred. Direct observation of the underlying atomic motions provides the full spectrum of motions, including so-called photodark states, missed with spectroscopic approaches.^4,7–9^ A direct observation of the key reaction modes directing solution phase chemistry captures the most relevant information and provides a highly visual means to understand the underlying reaction forces. The observed reaction dynamics is still convolved to the solvation coordinate in unknown ways. In this regard, one of the grand challenges of physical chemistry is to determine the role of the solvent in homogeneous chemistry. This objective requires the ability to prepare both gas phase and solution phase systems, under otherwise identical conditions, to study the molecular dynamics of interest with and without solvent. In the molecular beam community, progress has been made in this direction with the development of mass selected solvated clusters^26–28^ but so far has not been interfaced with structural probes to directly observe the difference in atomic motions. The number density of clusters is too low to enable sufficient signal to noise with present methodologies. Again, the sample delivery method is very sample specific and does not lend itself as a general method for the study of solution phase reaction dynamics.

To meet this challenge, the present work provides a simple, general method for solution phase sample delivery to the target region of a fs electron diffraction setup to atomically resolve molecular dynamics with and without solvent under identical conditions. The method is capable of injecting effectively any molecular system into the gas phase, up to systems as large as proteins, while simultaneously providing the same system in nanodroplets or liquid sheets to enable direct isolation of the effect of solvent on the molecular dynamics. The approach can be similarly applied to x-ray beamlines where there is less restrictions on the liquid thickness and would provide a more universal source for gas phase studies.

The method is based on the use of Picosecond InfraRed Laser (PIRL) pulses to create ablation conditions under full energy confinement, i.e., ablation faster than thermal or acoustic transport out of the excited volume.^29–31^ The mechanism involves the selective excitation of vibrational modes to deposit sufficient energy and associated heating rates to drive liquid to gas phase transitions faster than unarrested nuclei (bubble) growth that otherwise leads to cavitation, shock waves, and collateral damage or sample degradation.^29,32,33^ The relaxation of excited vibrational modes in the liquid state is universally faster than a few tens of picoseconds, by which the energy is redistributed into translational degrees of freedom, the very motions needed for ablation. For the energies used to drive this process, the degree of superheating, i.e., heating above the phase transition point, leads to a corresponding elevated lattice temperature that is well beyond the liquid to gas phase transition, for which the thermal motions completely overcome the binding potential of the liquid state. This time scale is much shorter than thermal diffusion or even acoustic propagation (thermal expansion) out of the excited volume. This mechanism is referred to as Desorption by Impulsive Vibrational Excitation (DIVE), where the term impulsive for the ablation process is used in relation to the speed of sound or acoustic propagation of energy out of the excited volume. The net effect is complete energy confinement to drive ablation for which the collective force is defined by the spatial profile for the absorbed energy and associated thermal gradient along the surface normal. This process leads to a nearly perfectly collimated ablation plume^29–31^ for which the uniformity of the forces involved leads to injection of molecules into the gas phase, proteins, even whole viruses, fully intact with no degradation.^34,35^ It has been shown that the fluorescence spectrum of Green Fluorescent Protein (GFP) is unaffected by the ablation process, which indicates that even conformational states are maintained due to the uniformity of the forces and the extremely short time scales involved in the ablation process.

To date, PIRL–DIVE has exclusively exploited the extremely strong absorption of water and universal presence in biological tissue to determine molecular composition in tissue, effectively providing a frozen snap shot of the entire composition from lipids to protein complexes.^36^ This mechanism has also been demonstrated as a new surgical tool capable of surgery at the single cell level with no collateral damage to the surrounding tissue.^33,37^ Given that this mechanism leads to ablation with conservation of complete molecular signatures for even complex tissues, this mechanism can be readily applied to other less heterogeneous systems. The study of homogeneous solution phase chemistry is just one important extension of this sample delivery concept. In this regard, all vibrational modes for liquids can be targeted to drive ablation. The differences from liquid to liquid will be in the absorption depth as determined by the oscillator strengths or molar absorptivities. The requirement to deposit sufficient energy to exceed the spinoidal point of the liquid/gas phase transition point can be simply accomplished by adjusting the excitation energy used to excite a given vibrational mode of the liquid.^29–32^ The leading edge of the resulting ablation plume is found experimentally from dark field and interferometric imaging to comprise of gas phase molecules moving at Mach 3 followed by nanodroplets that result from recoil effects from the displaced mass in the ablation plume.^29–31^ The composition of the leading edge of the ablation plume has been further confirmed by theoretical studies. The DIVE effect has been shown by high level atomistic MD calculations to give isolated gas phase molecules, in which the solvent is completely stripped from the guest solute molecules, even for molecules as large as proteins.^38^ For water, the trailing edge is clearly observed experimentally to give nanodroplets, which provides the sample delivery mechanism for solution phase systems.

The above features are particularly important for large molecules, which typically have very low vapor pressures and cannot be readily introduced into the gas phase for spectroscopic studies. The only other method capable of sample delivery of large molecules is electrospray,^39,40^ and the number densities are too low for most spectroscopic studies.^41^ In this regard, PIRL–DIVE represents a laser driven molecular beam with orders of magnitude higher number densities than electrospray or conventional molecular beams using differential pumping.^31,39,42,43^ In principle, any size molecule can be introduced into both gas phase and nanoscale liquid domains with the same sample delivery system. In this regard, the PIRL–DIVE method is truly unique.

We have expanded the use of PIRL–DIVE from water to glycerol by targeting the OH vibrational mode of glycerol for energy deposition. We chose glycerol primarily due to its similar solubility of most solutes to that of water and for its relatively low vapor pressure. This latter property enables the use of a simple microcapillary to bring the solvent into the vacuum environment of a femtosecond electron diffraction instrument. This choice of solvent system was also convenient as this absorption band lies within the spectral bandwidth of the PIRL laser system that has been optimized for the water OH absorption band. However, it is straightforward using parametric amplifiers to tune the PIRL excitation to any strongly absorbing absorption band for a given solvent to induce the DIVE process.

As will be shown below for certain PIRL excitation regimes, it is possible to generate nanosheets of glycerol under vacuum conditions as well as isolated gas phase molecules. Either gas phase or solution phase conditions can be selectively controlled by adjusting the time delay between pulse sequences and PIRL pulse energies. This new sample delivery concept makes it possible to use femtosecond electron diffraction to study reaction dynamics for both solution phase and isolated gas phase systems under effectively identical condition to directly observe and quantify the effects of solvent on reaction coordinates at the atomic level of detail.

## II. EXPERIMENTAL METHOD

**FIG. 1.**
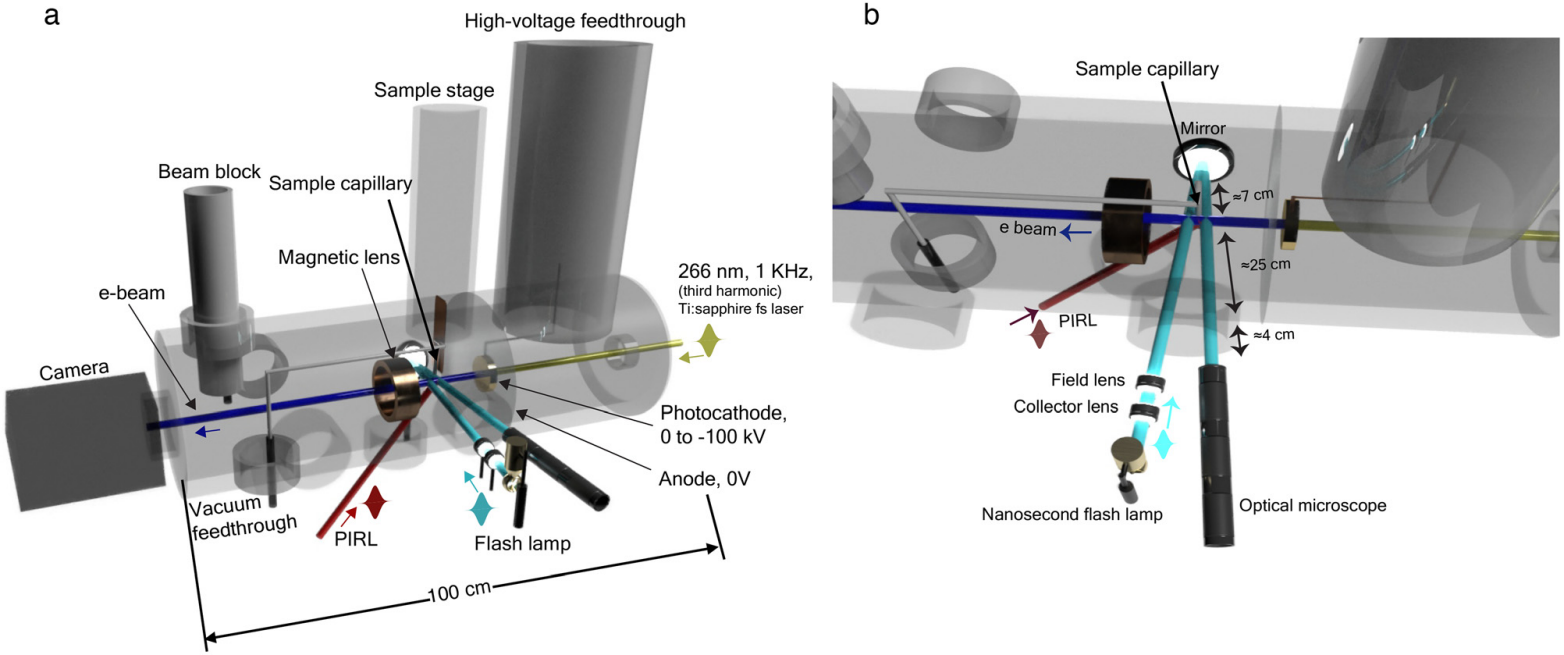
Schematic drawing of the experimental setup for coupling the PIRL–DIVE plume with a femtosecond electron gun and an optical bright-field microscope. (a) Schematic diagram of the side view of the experimental setup. (b) Schematic diagram of the top view of the experimental setup. See text for further details.

The setup is mainly constituted of three parts, i.e., the PIRL-driven sample source, the electron imaging part, and the optical imaging part. The first part is the PIRL-driven sample source. Here, we use glycerol as a prototype sample as it has a low vapor pressure and maintains the liquid state when injecting into vacuum. A sample reservoir was mounted on a height adjustable frame, which is close to the vacuum setup. The reservoir was connected by Teflon tubing to a stainless steel needle with an inner diameter of 0.45 mm, which was mounted on a three-axis sample translation stage inside the vacuum chamber. The flow of sample into the chamber was controlled by adjusting the height of the sample reservoir and back pressure connected to the reservoir to make sure there is liquid glycerol in the stainless-steel needle while avoiding liquid dropping into the chamber.^47^ The second part is the electron imaging part, which is shown in Fig. 1(a). The third harmonic of Ti:sapphire femtosecond laser (35 fs pulse duration at the amplifier output) was used to excite the photocathode, which is connected with a high-voltage feedthrough.^11^ The photocathode includes a 20 nm thin gold film, which produces pulsed photoelectrons under the UV fs laser pulse illumination. A grounded anode plate was mounted 8 mm away from the photocathode. The anode plate is attached to a flange, which enables differential pumping between the electron gun chamber and the sample source chamber. Two turbomolecular pumps were implemented to pump the two chambers, respectively. A magnetic lens was mounted between the sample and the detector. By adjusting the electric current, we can change the magnetic field strength of the magnetic lens, in another words, the focal length of the magnetic lens. Consequently, we can image the plume in both real space and reciprocal space by adjusting the magnetic lens current. The detector was a Princeton Instruments Quad-RO CCD fiber-optically coupled to a P43 scintillator. The third part is the optical imaging part, which is shown in Fig. 1(b). A discharge flashlamp pulsed source (Nanolite) with several nanoseconds pulse duration and broadband white light spectrum was used as the illumination source. A collector lens (f = 60 mm) collects the light initially, and then a field lens (f = 400 mm) focuses the beam. The optical illumination path was adjusted, so that the DIVE plume was in the field lens focal plane to achieve the maximum and uniform illumination.

An objective lens with a focal length of 300 mm was used to optically image the DIVE plume. A picosecond infrared laser (PIRL) from Light Matter Interaction Inc. with a 3 *μ*m central wavelength and 400 ps pulse duration (FWHM) was adopted as the ablation laser. The PIRL was focused on the sample by a 300 mm focal length lens. The focus spot size diameter (1/e^2^) is around 350 *μ*m. The PIRL pulse energy was adjusted by a series of neutral density filters to adjust the incident intensity to drive ablation in the range up to 1 J/cm^2^ focusing conditions.

The PIRL pulse acted as the master trigger. Both the flashlamp illumination source and femtosecond laser pulse for driving the photocathode were synchronized by a delay generator (Quantum Composer) using the timing signal from the PIRL pulse.^47^ The whole system was synchronized at the 1 kHz repetition rate. The delay between flashlamp and PIRL and femtosecond laser and PIRL could be adjusted by the delay generator. This set up allowed the imaging of the PIRL–DIVE plume under the same conditions using both electron and optical imaging. The ability to use electron diffraction in parallel allowed a direct determination of isolated gas phase and liquid phase components to the ablation plume. By adjusting the timing between the PIRL pulse and either the electron or pulsed optical source, it was possible to sample specific spatial regions of the plume. The time delay could be adjusted in 1 ns increments, which corresponds to micrometer resolution for a plume moving initially at Mach 3 as reported earlier.^30,31^ This spatial resolution is more than good enough as major changes in plume composition in relation to gas phase and liquid domain contributions were readily resolved with *μ*s time delays, as reported below. The chambers were evacuated by Pfeiffer turbomolecular pumps, which were pre-pumped by Edwards scroll pumps. The vacuum pressure of the sample source chamber during experiments is typically around 10^−6^ mbar, and the electron gun chamber is typically around 10^−7^ mbar. The effect of the liquid delivery system was negligible on the vacuum conditions needed.

One of the main attractive features of this approach is the very low sample consumption. Based on previous literature,^35^ a rough estimate for the sample ejection is 27 pl per shot. We need 2750 shots for producing a diffraction image with good signal to noise ratio, giving a sample consumption of 74.25 nl per diffraction image.^47^

## III. RESULTS AND DISCUSSION

### A. Imaging of gas-phase glycerol

The experimental setup allows optical imaging as well as use of electron pulses for real space and reciprocal space or diffraction imaging of the plume. The optical imaging of PIRL driven ablation plumes has been reported previously for both dark field and interferometric methods to increase contrast.^30,31^ In the present brightfield imaging, the optical images are determined by refractive effects that are related to density variations in the plume with the greatest contrast from nanodroplets for which scattering increases with increasing size. In the case of real space imaging with femtosecond electron pulses, the image contrast is related to the integrated number of molecules intercepted by the electron beam. Electron scattering cross sections at 100 kV result in penetration depths of less than 100 nm for liquid state densities, which corresponds to a few thousand intervening molecules within gas phase or liquid state. In the case of reciprocal space or diffraction imaging, the diffraction patterns can be uniquely related to either isolated gas phase molecules or liquid state by observation of the diffraction at wavevectors corresponding to the intermolecular distances in the bound liquid state. This multimodal imaging approach enables full characterization of the ablation plume with respect to spatial regions of isolated gas phase molecules and regions for which the energy distribution of the absorbed laser excitation was insufficient to overcome the intermolecular forces of the liquid state. This spatial relationship depends on the absorbed energy and degree of superheating above the liquid–gas phase transition, including the latent heat of vaporization.^29,30,32^

**FIG. 2.**
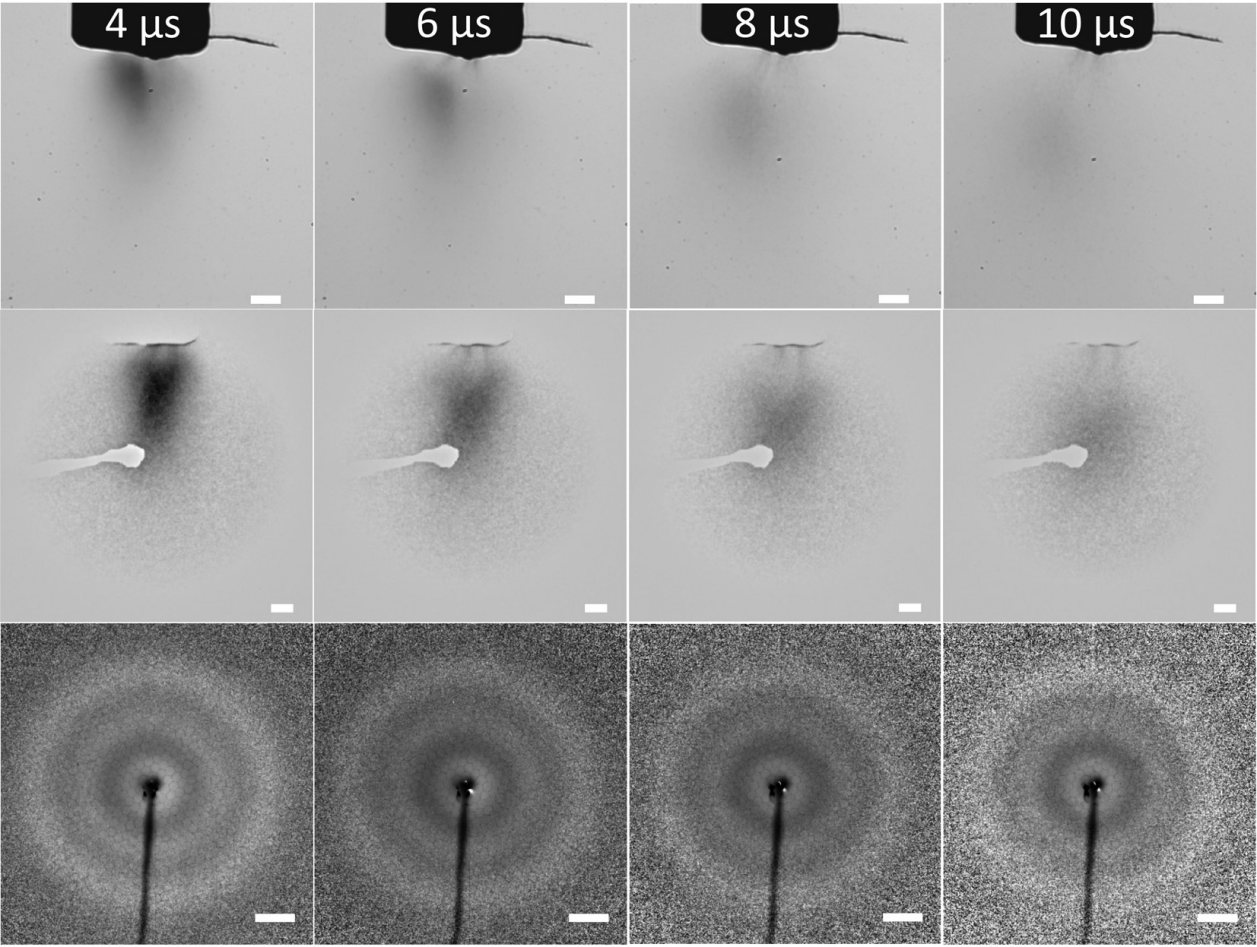
Upper row: 50 shots averaged optical images of PIRL-driven gas-phase glycerol plumes at different delays after the PIRL ablation pulse. The white scale bar corresponds to 200 *μ*m. Middle row: 550 shots averaged electron images of PIRL-driven gas-phase glycerol plumes in real space at different delays after the PIRL pulse. The white scale bar corresponds to 200 *μ*m. Bottom row: 2750 shots averaged electron diffraction images (I_*total*_ × s^2^) of PIRL-driven gas-phase glycerol plumes at different delays after the PIRL ablation pulse. The white scale bar corresponds to 20 nm^−1^. The PIRL fluence is at 450 mJ/cm^2^. See text for details.

The results for PIRL driven ablation of glycerol are shown in Fig. 2 for different time delays between the PIRL excitation and pulsed optical or electron imaging at 4, 6, 8, and 10 *μ*s to capture the plume formation and relative gas and liquid phase components. The upper row shows optical images for 50 shots averaged; the middle row shows electron real space images for 550 shots; and the bottom row shows electron diffraction for 2750 shots averaged using PIRL pulses with a peak fluence at 450 mJ/cm^2^. The gray scale contrast of the real space optical images and electron images reflects the density distribution of the plume, as discussed above. We can see from the real space optical and electron images that the plume has less density at longer delays since the plumes expand and become dilute. The electron diffraction images on these plumes are shown in the bottom row of Fig. 2. They were taken by changing the magnetic lens current from 840 mA for real space imaging to 490 mA to collimate the beam, for the smallest beam size at the detector plane, for diffraction imaging. We can clearly see the interference diffraction patterns. These diffraction patterns give direct information on the atomic structure of the isolated gas phase molecules within the plume as well as intermolecular spatial correlations related to liquid state components to the plume, at the different time delays, within the approximate 100 *μ*m spot size of the electron probe beam (FWHM). This information is given by the radial distribution of the diffraction pattern discussed below.

Based on previous work of imaging the PIRL–DIVE process in water, the plume is expected to be moving at approximately Mach 3, such that these intervening 2 *μ*s time delays correspond to points in space that would be separated by approximately 2 mm in the propagation direction.^29–31^ The plume at this excitation energy is comprised of mostly gas phase glycerol as determined from the diffraction patterns, so it is difficult to see a well-defined ablation front as is possible in air by virtue of the formation of a shock front in air.^29–31^

### B. Imaging of liquid-phase glycerol

**FIG. 3.**
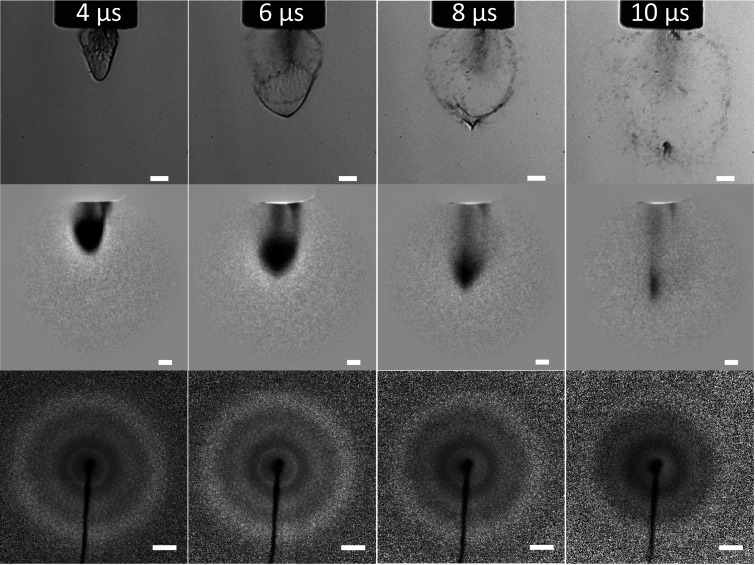
Upper row: single-shot optical images of the PIRL-driven glycerol thin liquid film (bubble) at different delays after the PIRL ablation pulse. The white scale bar corresponds to 200 *μ*m. Middle row: 550 shots averaged electron images of the PIRL-driven glycerol thin liquid film (bubble) in real space at different delays after the PIRL pulse. The white scale bar corresponds to 200 *μ*m. Bottom row: 2750 shots averaged electron diffraction images (I_*total*_ × s^2^) of glycerol thin liquid film (bubble) at different delays after the PIRL ablation pulse. The white scale bar corresponds to 20 nm^−1^. The PIRL fluence is at 220 mJ/cm^2^. See text for details.

Figure 3 shows single-shot optical images (upper row) and averaged electron images of glycerol plumes both in real space (middle row) and reciprocal space (bottom row) ablated by PIRL with a peak fluence at 220 mJ/cm^2^. From the single shot optical imaging (upper row of Fig. 3) results, we can clearly see that the glycerol bubbles were produced before 4 *μ*s. These bubbles expand during the ablation launch and effectively burst, with expansion forces overcoming surface tension, at 10 *μ*s after the PIRL pulse. The bursting event originates from the bottom of the bubbles in the reference frame of the optical images shown in Fig. 3. The middle row of Fig. 3 shows the corresponding electron images of the glycerol plumes in real space. We note here that multiple shots were needed for electrons relative to the optical case to makeup for the much smaller number of electrons relative to photons used in the different imaging modalities, with similar detector efficiencies, to arrive at similar image quality. The much higher scattering cross section of the electrons, however, gives a higher contrast to changes in molecular density. We can distinguish from both the optical and electron images that the bubble is fairly uniform by inspection, and the surface constitutes a continuous thin liquid film. The film must be on the order of 100 nm thick or less; otherwise, it would be opaque to the electron probe. From the optical images, the well-formed surface layer is observable and implies liquid density. We can see from the real space electron imaging results that the plume, indeed, has higher density at its bottom. This region begins to become thin from 4 to 6 *μ*s as the electron transmission increases near the bottom as can be seen comparing the optical and electron real space images. There is a very distinct v-neck that occurs at the bottom of this laser driven jet that is clearly observable at 6 and 8 *μ*s in the optical image. It is also visible in the electron image but not so well resolved due to the much higher absorption of electrons relative to the optical imaging and some blurring from multiple shots. The degree of agreement between single shot optical imaging and multiple shot averaging of electron images shows that this bubble or jet formation is quite regular or well defined. The observed v-neck formation of the surface is attributed to the elastic restoring force of the surface of the jet as it expands and the initial Gaussian profile for the PIRL energy deposition in the glycerol. This strong curvature and associated potential energy gradient are sufficient to burst the thin liquid film to create a gas jet.

In comparing the plume front for the different time delays, the plume appears to be moving at approximately the speed of sound (Figs. 2 and 3). However, between 6 and 8 *μ*s (Fig. 3), the recoil effect observable in the v-neck formation of the ultrathin liquid retards further the advance of the laser driven molecular beam. After the bubble surface bursts, the molecular beam accelerates and is effectively slingshot forward to recover the spatial position it would have had prior to the surface retardation effect.

**FIG. 4.**
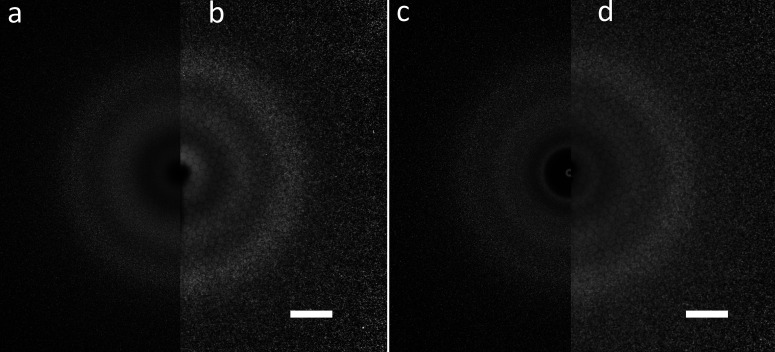
Simulated (a) and (c) and experimental (b) and (d) electron diffraction results (I_*total*_ × s^2^) on gas-phase glycerol (a) and (b) and liquid-phase glycerol (c) and (d). The white scale bar corresponds to 20 nm^−1^. See texts for details.

The ability to conduct electron diffraction under the same conditions allows the determination of atomic structure of the molecules forming a gas phase expansion as well as any spatial correlations that might be attributed to nanoscale liquid domains. In this regard, the electron diffraction images on these plumes are shown in the bottom row of Fig. 3. We can see the interference diffraction patterns from the liquid glycerol thin films directly. Compared with the diffraction patterns of gas-phase glycerol shown in the bottom row of Fig. 2, there is a sharp bright ring that now appears at the low scattering sector regime. This diffraction ring in reciprocal space corresponds to new spatial correlations at larger interatomic distances than the C–O and C–C bonds of glycerol. It should be assigned to intermolecular diffraction involving distinct, periodic, and spatial relationships or atom pair correlations between molecules. The specific scattering vector is a signature of a liquid diffraction peak and matches quite well with the theoretical calculation shown in Fig. 4(d).

### C. Theoretical calculation

Here, we calculated the expected electron diffraction observable for both gas and liquid phase glycerol. The two will differ by the additional intermolecular spatial correlations due to the intermolecular forces present for the liquid state. In this regard, the measured experimental total electron scattering *I_tot_* of the DIVE plume contains atomic scattering *I_atom_*, molecular scattering *I_mol_*, and experimental background *I_bg_*, which includes inelastic scattering, multiple scattering, and system-specific background.^44^

The contribution of the atomic scattering to the overall scattering intensity is simply given as the sum of all the elastic scattering amplitudes for all atoms in the system,

$$I_{\mathrm{atom}}(s)=\sum_{i=1}^{N}|f_{i}(s){|}^{2},$$ (1)

where s is the scattering vector, s=(4π/λ) sin (θ/2), N is the number of atoms in the system, and $f_{i}(s)$ is the elastic scattering amplitude for the *i*th atom.

The molecular scattering term can be expressed as a sum of interference terms for all atom pairs in the system, and the molecular scattering intensity *I_mol_* is given as follows:^44,45^

$$I_{\mathrm{mol}}(s)=\sum_{i=1}^{N}\sum_{j\neq i}^{N}|f_{i}(s)||f_{j}(s)|\;\text{cos}\;(\eta_{i}-\eta_{j})\frac{\;\text{sin}\;(s\times r_{ij})}{s\times r_{ij}},$$ (2)

where $f_{i}(s)$ and $f_{j}(s)$ are the elastic scattering amplitudes of the *i*th and *j*th atom, respectively, and *η_i_* and *η_j_* are their corresponding phases. *r_ij_* is the internuclear distance between the *i*th and *j*th atoms.

Figure 4 shows the comparison between the experimental and theoretical calculated diffraction using the known structure of glycerol and its liquid state. The theoretical results are calculated from the sum of atomic scattering and molecular scattering then adding up the Poisson noise. Figure 4(a) shows the electron diffraction theoretical result of gas-phase glycerol. Figure 4(b) shows the electron diffraction experimental results of gas-phase glycerol plumes produced by PIRL under the fluence at 450 mJ/cm^2^ with a delay between the electron pulse and PIRL pulse at 4 *μ*s. Figure 4(c) shows the electron diffraction theoretical result of liquid-phase glycerol. Figure 4(d) shows the electron diffraction experimental results of thin-liquid glycerol films (bubbles) prepared by PIRL under the fluence at 220 mJ/cm^2^ with a delay between the electron pulse and PIRL pulse at 4 *μ*s. From these results, we can clearly see the difference between the gas-phase and liquid-phase glycerol diffraction. There is an extra sharp constructive diffraction ring, at approximately 1.6 Å^−1^, in the inner part of the images for thin liquid glycerol film (bubble) diffraction, which is absent in the gas-phase glycerol diffraction. This feature is located at the low q range, which corresponds to the large interatomic distances and associated atom pair correlations involving C and O atoms predominantly. This feature was also observed in liquid glycerol neutron diffraction experiments^46^ and demonstrates that this electron diffraction pattern corresponds to glycerol in the liquid state.

**FIG. 5.**
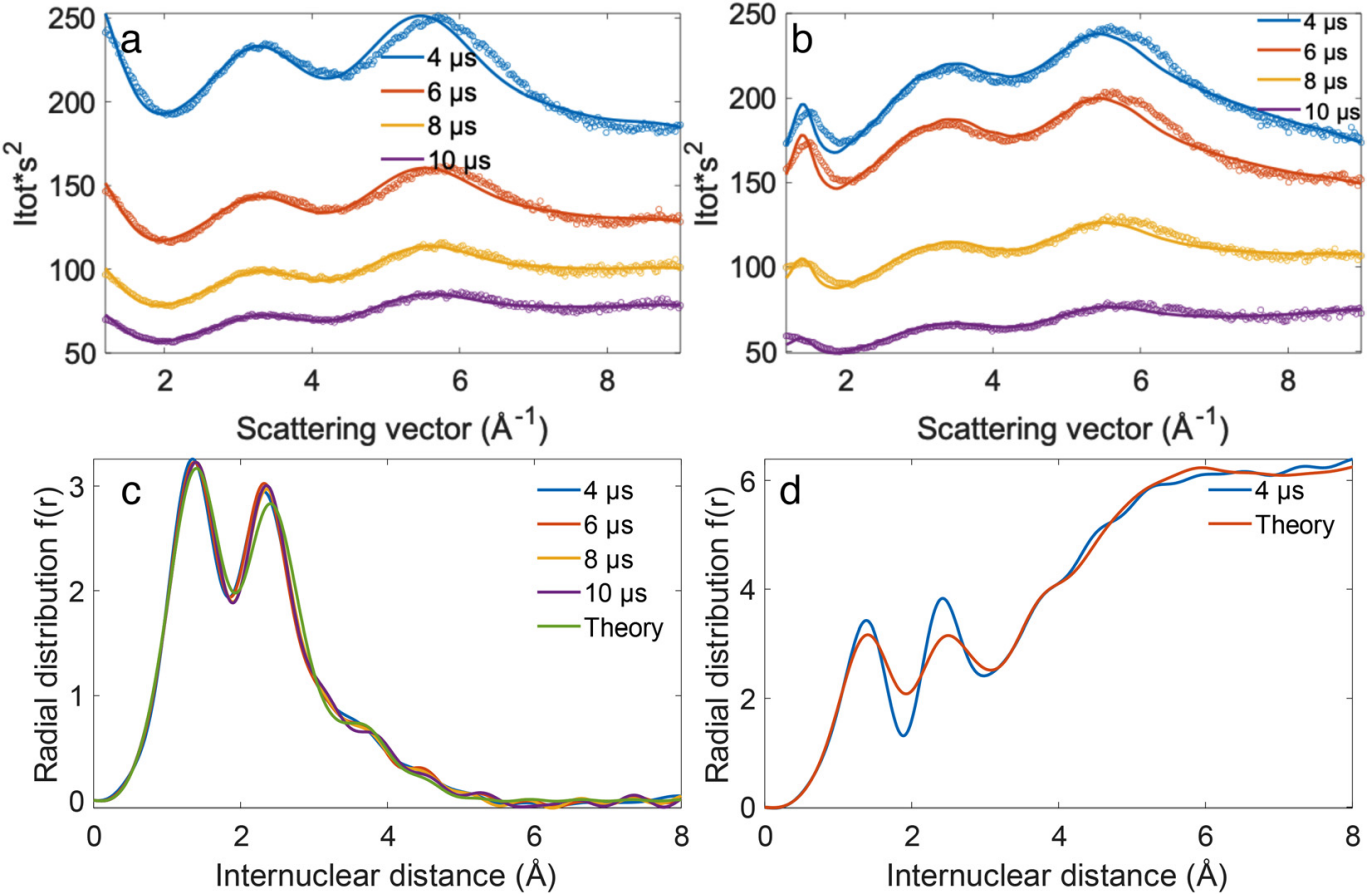
Radial average curves of two-dimension electron diffraction patterns from PIRL-DIVE plumes prepared with a PIRL fluence of 450 mJ/cm^2^ (a) and 220 mJ/cm^2^ (b). The dash lines are the experimental results. The solid curves are the fitted results. The panels (c) and (d) are the corresponding radial distribution curves f(r) obtained by sine transforming the radial average curves of (a) and (b), respectively. See text for details.

Figure 5 shows the radial averages of the two-dimension electron diffraction results of gas-phase glycerol plumes and liquid-phase glycerol plumes under PIRL fluence at 450 and 220 mJ/cm^2^, respectively. The solid curves are the calculated results for gas phase and liquid phase glycerol. For reference, the peaks at approximately 5.7 and 3.3 Å^−1^ in both Figs. 5(a) and 5(b) are contributions from constructive interference of an electron plane wave diffracted by the glycerol atoms. At the lower excitation conditions, insufficient to completely vaporize glycerol [Fig. 5(b)], we can clearly see the peak of the intermolecular atomic pair contribution for the liquid-phase glycerol at 1.6 Å^−1^. As the glycerol bubble contains both gas-phase and liquid-phase glycerol, the contribution for gas-phase and liquid phase can be disentangled by fitting the experimental results with both the theoretical gas-phase and liquid-phase electron diffraction results. The fitting results are shown in Table I for the 220 mJ/cm^2^ excitation condition. It shows that the contribution of gas-phase and liquid-phase varies with the delay between electron pulses and PIRL pulses. The composition changes most significantly to gas phase upon the breakup of the bubble that occurs between 8 and 10 *μ*s time delays. This change corresponds to the loss of the liquid phase and associated intermolecular distribution defining the surface of the bubble that converts to gas phase upon collapse. This feature is interesting as the excitation at 220 mJ/cm^2^ is at the threshold to overcome the latent heat of vaporization, i.e., providing sufficient energy to overcome the liquid surface tension. We are observing the dynamic coupling and exchange of energy between the translationally hot glycerol gas phase molecules and the confining liquid layer defining the surface tension. The forces leading to expansion and bubble formation likely come from the collective acoustic forces that would result from rapid thermal expansion and recoil from the unexcited liquid boundary. This thermally driven acoustic wave would act to displace the liquid surface area and drop the pressure causing the transition from liquid to gas phase as part of the observed bubble formation and ultimately its collapse. This rapid phase transition and co-existence of liquid and gas phase are interesting phenomena that merit further study in terms of understanding strongly driven nonequilibrium phase transitions with respect to rapidly changing thermal and pressure variables.

**TABLE I.** Proportion of gas-phase and liquid-phase glycerol samples in the thin-liquid bubble film.

Delay (*μ*s)	Liquid-phase contribution (%)	Gas-phase contribution (%)
4	39	61
6	41	59
8	35	65
10	26	74

### D. Atomic structure determination

The radial distribution curve *D*(*r*) that represents the sum of probabilities of two nuclei separated by distance *r* can be derived by sine transforming the modified molecular scattering data,^44,45^

$$D(r)=\int_{0}^{\infty }sM(s)\times \text{sin}\;(s\times r)ds,$$ (3)

where *sM*(*s*) is the modified molecular scattering,

$$sM(s)=s\frac{I_{\mathrm{Mol}}(s)}{I_{\mathrm{atom}}(s)}.$$ (4)

Since the scattering vector *s* in the measured experimental data is finite, there is insufficient signal to noise to merit taking the integration to infinity. The modified radial distribution *f*(*r*) is usually used,

$$f(r)=\int_{0}^{s_{\text{max}}}sM(s)\times \text{sin}\;(s\times r)e^{-ks^{2}}ds,$$ (5)

where $e^{-ks^{2}}$ is a Gaussian window function to account for the cutoff of *s* at *s_max_*. The cutoff at low *s* is appended by the theoretically derived *sM*(*s*).

Figures 5(c) and 5(d) show the radial distribution curve of glycerol ablated under PIRL fluence at 450 and 220 mJ/cm^2^. The two main peaks at around 1.4 and 2.4 Å correspond to the bonded C–O distance and non-bonded intramolecular C–O distance which matches with the theoretical data well, respectively.^47^ The increasing amplitude in the radial distribution for distances beyond 3 Å in Fig. 5(d) is due to the distribution of intermolecular distances in the liquid state that is clearly missing in Fig. 5(c), assigned as predominantly gas phase diffraction. This distinguishing feature is consistent with the sharp peak at low *s* regime as assigned for liquid glycerol.

## IV. CONCLUDING REMARKS

A laser-driven molecular source that delivers both gas-phase and thin liquid film samples was coupled with an ultrafast electron gun and a bright-field optical microscope. This approach enabled imaging the prepared plumes with electrons and photons simultaneously. By varying the magnetic lens current for the electron source, we can image the plumes in real space and reciprocal space, with the latter providing atomic information on the plume composition. This table-top setup provides a simple approach to simultaneously conduct gas-phase and liquid-phase electron and optical imaging of molecular dynamics. The results demonstrate that the PIRL–DIVE driven molecular plume can deliver both gas-phase and liquid-phase samples into the electron interaction points for femtosecond electron diffraction studies. By comparing the experimental diffraction results with theoretical results, we can disentangle the contribution from liquid-phase and gas-phase samples. This paves the way to study ultrafast reaction dynamics of molecules under different solvation conditions. As the sample source, electron source, and optical illumination source are all pulsed and synchronized, this setup significantly reduces the sample consumption and gives essentially 100% hit rates based on the highly reproducible plume images. The ability to rapidly introduce new samples in perfect synchronization for stroboscopic time resolved studies eliminates problems in accumulated radiation damage between pulses from either laser excitation or electron probe pulses. The other important feature of this work is that the PIRL–DIVE process makes it possible to inject effectively any molecular system into the gas phase up to the size of proteins, even whole viruses, that are otherwise difficult, if not impossible, to get into the gas phase with sufficient density for electron diffraction determination of structure and dynamics. We note here that given the approximate 10 *μ*s time for plume transit through the probed region, the sampling rate could be increased from 1 to 10 KHz for signal averaging to increase the signal to noise to improve spatial resolution.

The most unique feature of this work is that it provides a means to selectively study virtually any system of interest for both fully solvated and isolated gas phase conditions under effectively identical conditions. This work makes it possible to compare with and without solvent to directly determine the effect of the solvation coordinate on molecular dynamics—one of the longest standing problems in physical chemistry. With sufficient coherent electron sources, it may also be possible to unveil mesoscale structures and conformational dynamics of biomolecules without the need for crystals and avoid crystal contact effects on protein conformational dynamics. Within the spatial resolution limits for liquid phase diffraction, it is now possible to study all classes of nonreversible light induced reaction dynamics and molecular dynamics of mesoscale, biological systems, simultaneously in gas-phase and liquid phase to reveal the secrets of solvation effects.

## Data Availability

The data that support the findings of this study are available from the corresponding author upon reasonable request.
